# Incidental Acute Appendicitis on Fluorine-18-Fluorodeoxyglucose Positron Emission Tomography/Computed Tomography (18F-FDG PET/CT) Imaging: Radiology From a Different Perspective

**DOI:** 10.7759/cureus.17734

**Published:** 2021-09-05

**Authors:** Samar Hamid, Sadaf Nausheen, Naveed Ahmed, Riffat P Hussain, Nida Ihsan

**Affiliations:** 1 Radiology, Jinnah Postgraduate Medical Centre, Karachi, PAK; 2 Cyberknife Robotic Radiosurgery, Jinnah Postgraduate Medical Centre, Karachi, PAK; 3 Nuclear Medicine, Jinnah Postgraduate Medical Centre, Karachi, PAK

**Keywords:** acute appendicitis, increased fdg uptake in appendix, hybrid imaging, classic hodgkin's lymphoma, 18f-fdg pet/ct

## Abstract

Although Fluorine-18-Fluorodeoxyglucose Positron Emission Tomography/Computed Tomography (18F-FDG PET/CT) is routinely used in oncological imaging, the F-18 fluorodeoxyglucose (18F-FDG) avidity is not tumor-specific. Numerous benign infective and inflammatory processes may also show increased radiotracer activity. Similarly, abnormal 18F-FDG uptake in an inflamed appendix can pose a diagnostic challenge for the interpreter of oncologic 18F-FDG PET/CT. We present the case of an 18-year-old female with classic Hodgkin's lymphoma who had 18F-FDG PET/CT while undergoing chemoradiotherapy. The scan demonstrated a complete metabolic response to treatment. However, there was increased 18F-FDG uptake in the right iliac region, projecting over the appendix, which, if interpreted as a lymphomatous involvement, would have upscaled the treatment response to progressive disease. The patient was called for additional workup, which included an ultrasound abdomen.

The scan revealed classic features of acute appendicitis. However, there was no appendicolith or luminal obstruction. Upon additional questioning, the patient mentioned mild intermittent abdominal pain and anorexia eased by pain relievers for the preceding few days. On deep palpation of her abdomen, there was rebound tenderness in the right iliac region. According to the Alvarado score, it was graded 7 points suggesting probable/likely appendicitis. After collective evaluation of the clinical, laboratory, and imaging findings, the appendicular 18F-FDG uptake was deemed secondary to uncomplicated acute appendicitis rather than a lymphomatous lesion. Our patient refused surgery as she did not have severe abdominal pain. She was hemodynamically stable without signs of luminal obstruction. She was non-operatively managed with broad-spectrum antibiotics for six days. The results of the follow-up complete blood counts and ultrasound examination were negative. Our patient was symptom-free and recovering normally at a two-week follow-up appointment.

We present a follow-up case of classic Hodgkin's lymphoma with incidental uptake in the appendix, which resembled submucosal lymphomatous cell infiltration of the appendix. Careful scrutiny, clinical correlation, physical examination, blood tests, and additional imaging offered helpful insight and led to the correct, benign diagnosis of the 18F-FDG avid appendix.

## Introduction

Fluorine-18-Fluorodeoxyglucose Positron Emission Tomography/Computed Tomography (18F-FDG PET/CT) has revolutionized oncological imaging, and hence it is fundamental to understand its limitations for accurate interpretation. Hybrid imaging is regularly utilized worldwide to diagnose, stage, restage, and follow up different malignancies [1]. Even if no apparent morphology is seen on anatomical imaging, the 18F-FDG PET/CT scan reveals abnormal metabolic uptake at the molecular level [2,3]. 18F-FDG is the most abundantly used radiotracers in oncologic imaging owing to its similar behavior to glucose and hence tissue accumulation in proportion to its level of glycolysis. Unfortunately, 18F-FDG is not particular to cancer, and it can accumulate in a tissue for a variety of reasons, the most predominant of which is inflammation [4]. Any focus of inflammation can appear on an 18F-FDG PET/CT scan as a hypermetabolic area. Therefore, the interpreter must consider benign pathologies unrelated to the underlying primary neoplastic process to avoid wrongly upstaging or downstaging the disease [5].

Acute appendicitis is one such condition in which profound inflammation develops due to luminal obstruction by fecolith [6]. It is this element of inflammation that brightens up on an 18F-FDG PET/CT scan. Acute appendicitis is diagnosed primarily by ultrasound and clinical examination. The characteristic sonographic features include a blind-ended tubular structure with mural thickening and an increased caliber of more than 6 mm in the right iliac region [7]. The CT scan usually shows increased appendiceal caliber with periappendiceal haziness, thickening of the lateral conal fascia, adjacent free fluid, enlarged lymph nodes, and appendicolith [6]. 18F-FDG PET/CT is not indicated for evaluating acute appendicitis, yet, it may be diagnosed incidentally during the workup of malignant diseases. An inflamed appendix may show increased 18F-FDG uptake due to the underlying inflammation. There are few reported cases of acute appendicitis in the literature that were incidentally detected on 18F-FDG PET/CT [5-15]. We present the case of an 18-year-old girl who underwent 18F-FDG PET/CT to evaluate treatment response to chemoradiotherapy for classic Hodkin's lymphoma and was incidentally diagnosed with uncomplicated acute appendicitis.

## Case presentation

An 18-year-old female undergoing treatment for classic Hodgkin's lymphoma visited the PET/CT facility of Jinnah Postgraduate Medical Centre, Karachi, Pakistan, for a follow-up 18F-FDG PET/CT scan. Her diagnosis was established based on the histopathology of a right cervical node. Her baseline 18F-FDG PET/CT scan demonstrated enlarged hypermetabolic nodes in the right cervical region, which were treated with six cycles of chemotherapy (adriamycin, bleomycin sulfate, vinblastine sulfate, and dacarbazine) followed by four fractions of radiotherapy to the cervical region. At the time of her follow-up 18F-FDG PET/CT scan, a dose of 165.76 MBq of F-18 FDG was administered through the right hand. The scan was performed after a delay of 60 minutes. Initially, molecular imaging was performed from the head to mid-thighs. A low dose plain CT scan was then obtained over the same region for anatomic correlation and attenuation correction. 

**Figure 1:**
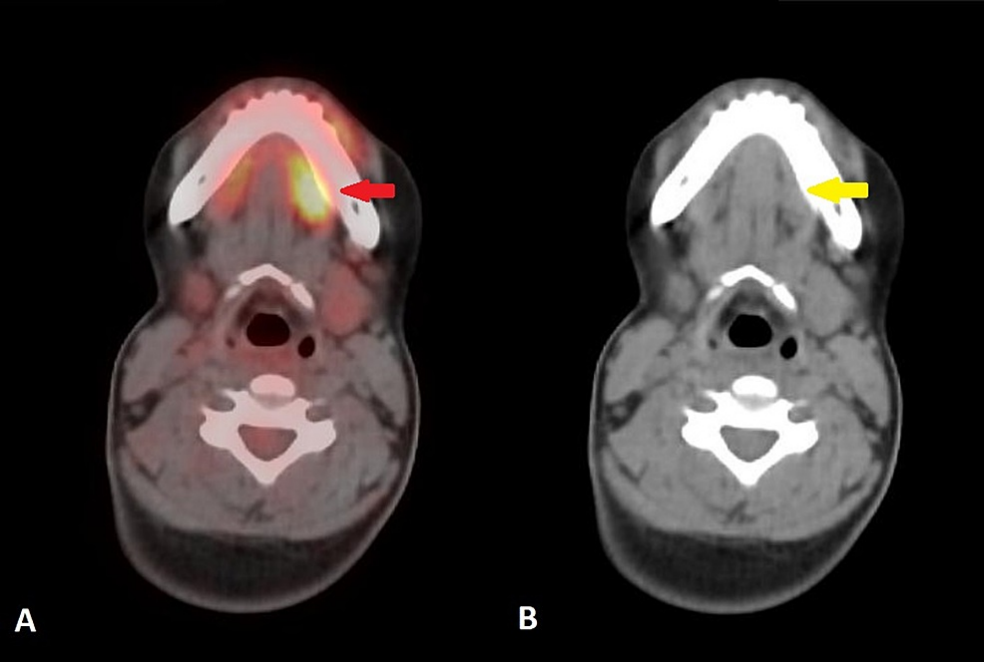
18F-FDG PET/CT axial images of head and neck region (A) fused image (B) CT image (A) Fused image shows linearly orientated 18F-FDG uptake in the floor of the mouth, projecting over the left mylohyoid (red arrow) (B) CT image shows normal appearance of the left mylohyoid muscle (yellow arrow) without any soft tissue mass or enlargement. These findings represent physiologic uptake. 18F-FDG PET/CT: Fluorine-18-Fluorodeoxyglucose Positron Emission Tomography/Computed Tomography

**Figure 2:**
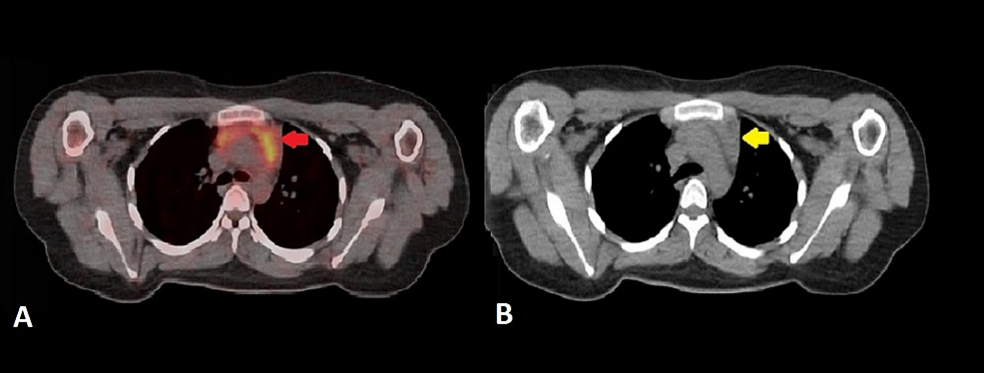
18F-FDG PET/CT axial images of upper chest and mediastinum (A) Fused image shows diffuse 18F-FDG uptake in the anterior mediastinum projecting over the thymus (B) CT image shows moderately enlarged thymus. Keeping the patient's recent history of chemotherapy, these findings represent rebound thymic hyperplasia. 18F-FDG PET/CT: Fluorine-18-Fluorodeoxyglucose Positron Emission Tomography/Computed Tomography

The baseline and interim 18F-FDG PET/CT scans were compared to the current examination in detail on the Mirada workstation (Mirada Medical, Oxford, UK). The previously seen 18F-FDG avid cervical nodes had resolved entirely in the current scan. There was no evidence of any newly developed 18F-FDG avid node in the neck, chest, or abdomen. There was no evidence of pleuropulmonary, hepatic, adrenal, splenic, or skeletal lymphomatous involvement. A few areas of benign/nonspecific uptake were noted in the 18F-FDG PET/CT scan. In the head and neck region, there was newly developed linearly orientated 18F-FDG uptake in the floor of the mouth, projecting over the left mylohyoid muscle, without anatomical correlate suggesting physiologic uptake (Figure 1). In the mediastinum, there was diffuse 18F-FDG uptake in the moderately enlarged thymus, which considering the history of chemotherapy, was regarded as post-chemotherapy rebound hyperplasia (Figure 2). Mild 18F-FDG avid tonsillar enlargement was also noted.

**Figure 3:**
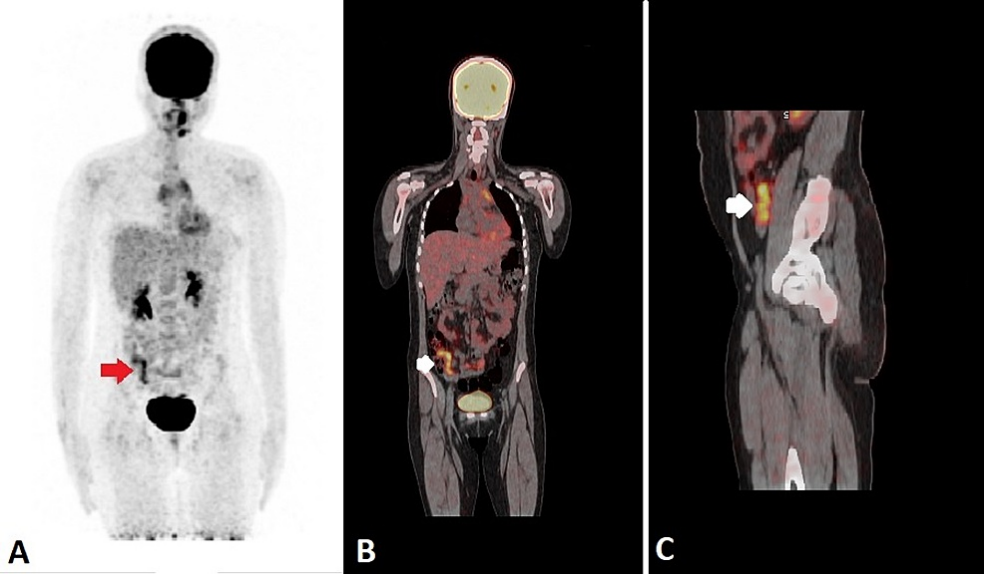
18F-FDG PET/CT: (A) coronal MIP image, (B) coronal fused image and (C) sagittal fused image (A) MIP image shows an 18F-FDG avid finger-like structure (red arrow) in the right iliac region. (B) Coronal fused image shows a hypermetabolic finger-like structure (white arrow) in the right iliac region. (C) Sagittal fused image shows hypermetabolic inflamed appendix (white arrow) lying anterior to the right psoas muscle. MIP: maximum intensity projection 18F-FDG PET/CT: Fluorine-18-Fluorodeoxyglucose Positron Emission Tomography/Computed Tomography 18F-FDG: F-18 fluorodeoxyglucose

**Figure 4:**
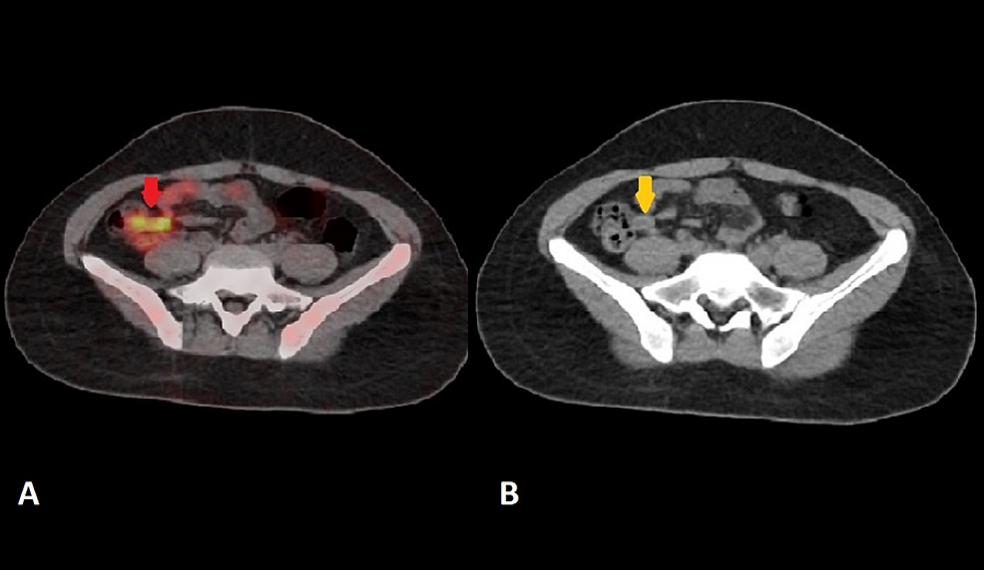
18F-FDG PET/CT axial images: (A) fused image (B) CT image (A) Fused image shows 18F-FDG avidity projecting over the appendix (red arrow) (B) CT image shows a fluid-filled dilated appendix with mural thickening (yellow arrow). No evidence of periappendicular fat stranding 18F-FDG PET/CT: Fluorine-18-Fluorodeoxyglucose Positron Emission Tomography/Computed Tomography

Alarmingly, there was a new focus of 18F-FDG uptake (maximum standardized uptake value [SUVmax] 5.5) in the right iliac region, which imitated the shape of a finger-like structure connected to the medial wall of the cecum. On correlative CT images, the appendix appeared dilated with diffuse wall thickening of up to 6.0 mm (Figures 3, 4). There was no evidence of adjacent fat stranding. Unexpected and incidental foci of uptake in oncologic 18F-FDG PET/CT are relatively common with diagnostic considerations, including unusual sites of metastases, a second synchronous neoplasm, post-surgical changes, and infection and inflammation. In our patient, the top differentials for appendicular 18F-FDG uptake included either submucosal infiltration of the appendix by lymphomatous cells or acute appendicitis. Despite the absence of 18F-FDG uptake everywhere in the body, the appendicular uptake was alarming since it indicated progressive disease rather than a complete metabolic response. This shift in the magnitude of the metabolic response to treatment had significant implications for subsequent management and treatment planning. Hence, the 18F-FDG avidity in the right lower quadrant warranted careful interpretation to establish the metabolic response to chemotherapy and avoid misdiagnosis of the lymphomatous spread of disease.

**Figure 5:**
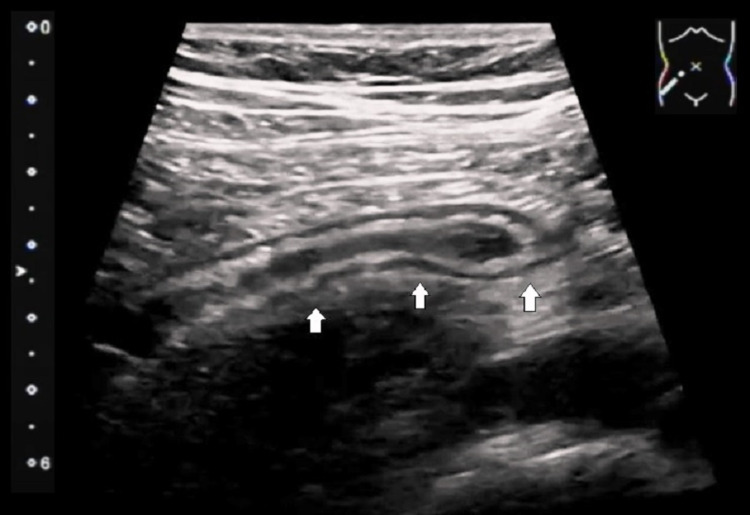
Longitudinal ultrasound image of the right lower quadrant An inflamed appendix (white arrows) in the right lower quadrant giving the shape of a dilated blind-ended tubular structure with mural thickening and mild periappendiceal fluid

The patient was requested to revisit the hospital for further evaluation. Before the 18-F FDG PET/CT scan, our patient did not mention any active complaints at the time of clinical evaluation. On directed questioning, she mentioned intermittent right lower quadrant pain for the past few days, relieved by painkillers. She also reported anorexia for the past two days. An abdominal ultrasound was performed the same day, which demonstrated an inflamed appendix in the right iliac region, giving the shape of a blind-ended tubular structure with mild periappendiceal fluid. There was no evidence of hyperechoic focus within the lumen casting posterior acoustic shadowing to suggest the presence of an appendicolith (Figure 5). Her laboratory tests revealed leucocytosis of 12700/μl with 80% neutrophils, serum hemoglobin 12.1 g/dl, platelets 350,000/mcL, and C-reactive protein 45.0 mg/dl. The consecutive serum lactose dehydrogenase levels during the past three months were 272.0 U/L, 251.0 U/L, and 242.0 U/L. On physical examination, there was rebound tenderness in the right iliac region on deep palpation. According to the Alvarado score, it was graded 7 points suggesting probable/likely appendicitis. After collective evaluation of the clinical, laboratory, and imaging findings (ultrasound), the appendicular 18F-FDG uptake was deemed secondary to uncomplicated acute appendicitis rather than lymphomatous involvement of the appendix. Our patient refused surgical intervention as she did not have severe abdominal pain. She was hemodynamically stable and was treated with broad-spectrum antibiotic therapy (ceftriaxone 2 grams once a day for six days). The follow-up complete blood counts were: white blood cells 6800/μl with 55% neutrophils, hemoglobin 11.5 g/dl, platelets 315,000/mcL, and C-reactive protein 8.0 mg/dl. The follow-up ultrasound examination was negative. On a two-week follow-up visit, our patient was utterly symptom-free with an uneventful recovery.

## Discussion

The lifetime risk of acute appendicitis is between 6% and 9%, with 86 cases per 100,000 people [9]. As a result, there are only a few cases of appendicitis in current literature that were incidentally imaged and diagnosed on 18F-FDG PET/CT. Most reports described symptomatic patients who underwent 18F-FDG PET/CT to evaluate a suspicious abdominal mass. There was 18F-FDG uptake in all of these right lower abdominal masses with metabolic uptake ranging from SUVmax 7.27 to 22.0. These patients were treated surgically, and the surgical specimens came out to be inflamed appendices with surrounding mesenteric fat inflammation [2,6,8,10].

Appendicitis was incidentally discovered on 18F-FDG PET/CT imaging during restaging workup of a squamous cell carcinoma of the head and neck region (SUV 4.3). The histopathology revealed subacute inflammation on a background of chronic appendicitis [5]. Park et al. described a rare cause of appendicitis in a patient with small-cell lung carcinoma. This patient developed submucosal invasion of the appendix by the metastatic cells resulting in luminal obstruction, inflammation, and perforation [11]. 18F-FDG avid inflamed appendix (SUV 6.9) was identified on an 18F-FDG PET/CT. This sort of luminal obstruction may take several days to occlude the lumen completely, and hence patients may remain asymptomatic for a long time [6,8,10]. Koff et al. reported a patient with a testicular, metastatic germ cell carcinoma who had asymptomatic 18F-FDG uptake (SUV 6.9) in the appendix on post-treatment follow-up 18F-FDG PET/CT. A laparoscopic appendectomy was performed, which showed acute suppurative appendicitis. This case exemplified the relevance of incidental findings detected on 18F-FDG PET/CT [12].

Ogawa et al. shared the experience of two false-positive cases of 18F-FDG accumulation in the appendix, which were initially diagnosed as malignant appendicular masses. These patients presented with right lower abdominal pain. A palpable mass was noticed on clinical examination in the right iliac region. The blood chemistry reports of these patients were within a normal range. A contrast-enhanced CT showed an enlarged appendix with thickened walls, non-homogeneous enhancement, and extensive periappendicular fat stranding. These findings were suspicious for a malignant lesion, and hence hybrid imaging was employed. On 18F-FDG PET/CT, there was intense 18F-FDG uptake in both appendicular masses ranging from SUVmax 7.27 to 17.11, and these were diagnosed as malignant masses. The final diagnosis of appendicitis was established on histopathology, which showed ulcerations with prominent infiltration of inflammatory cells, macrophages, and pus cells in the appendix and surrounding fat [14].

An additional PET CT protocol, dual time point imaging, which involves obtaining a second delayed acquisition, has been proposed to improve the overall sensitivity and specificity of 18F-FDG PET CT to identify cancer and discriminate it from other benign 18F-FDG avid pathologies. Malignancies frequently show greater FDG uptake on the delayed images than infectious and inflammatory foci, which show lower uptake on the delayed imaging. However, the performance of this delayed protocol and its overall dependability and utility have been inconsistent, limiting its routine clinical application for analyzing incidental foci of uptake on 18F-FDG PET/CT [16]. The European Association of Nuclear Medicine practice guideline/Society of Nuclear Medicine and Molecular Imaging guidelines for the use of 18F-FDG PET/CT in infection and inflammation, published in 2013, do not encourage the use of dual time point imaging, citing low dependability in discriminating neoplasm from infection as a reason [17].

The underlying etiology for abnormal 18F-FDG uptake may be determined by the patient's medical history and symptoms. A complete evaluation of the patient's oncologic history, treatment, procedures, infections, locations of discomfort, and other acute medical issues should be obtained. After that, the 18F-FDG PET/CT should be evaluated based on the patient's oncologic history. Knowing the typical pattern of metastases for the cancer being investigated can be quite helpful. In addition to patient history, corresponding CT images may guide the interpreting physician to the correct etiology of the focus of 18F-FDG uptake. Therefore, the interpreting physician must be familiar with common CT imaging presentations of acute inflammatory conditions encountered incidentally. Regardless of these measures, there will be indeterminate foci of radiotracer uptake in the 18F-FDG PET/CT of oncology patients. In such circumstances, clinical correlation, concomitant use of other imaging modalities, immediate or short-term follow-up diagnostic imaging, or endoscopy are other available tools [18].

Acute appendicitis appears to be a rare event, based on the number of 18F-FDG PET/CT scans performed each year [9,10,15]. While 18F-FDG PET/CT is not utilized to evaluate suspected acute appendicitis, it may be encountered as an incidental or unsuspected finding [8]. Therefore, the interpreter should consider this rare possibility while reporting an oncologic 18F-FDG PET/CT scan. A detailed further workup, including clinical evaluation, blood tests, and ultrasonography, led to the diagnosis of uncomplicated acute appendicitis in the present case report. Additionally, the absence of appendicolith and luminal obstruction and lack of periappendiceal inflammation might be the underlying reasons for our patient's response to non-operative management. 

## Conclusions

Despite the fact that 18F-FDG PET/CT is widely used in oncology, 18F-FDG avidity is not tumor-specific. Various benign processes may also show increased radiotracer activity and mislead in hybrid imaging. Rarely an inflamed appendix can show increased 18F-FDG avidity, and interpretation must be made with caution to avoid misdiagnosing as a metastatic or malignant lesion. To reach a final diagnosis, the interpreter should consider the possibilities of infective and inflammatory etiologies and employ additional imaging modalities with detailed clinical evaluation. Occasionally further workup and follow-up imaging may also be required. Hence, from our case, we concluded that a nuclear physician must think outside the box and consider that abnormal 18F-FDG uptake could be caused by a pathology unrelated to the primary neoplastic disease.
